# The FOXM1–ABCC5 axis contributes to paclitaxel resistance in nasopharyngeal carcinoma cells

**DOI:** 10.1038/cddis.2017.53

**Published:** 2017-03-09

**Authors:** Youxiang Hou, Qianling Zhu, Zheng Li, Yongbo Peng, Xiaohui Yu, Bowen Yuan, Yijun Liu, Youhong Liu, Linglong Yin, Yuchong Peng, Zhenghua Jiang, Jinping Li, Bowen Xie, Yumei Duan, Guolin Tan, Kurban Gulina, Zhicheng Gong, Lunquan Sun, Xuegong Fan, Xiong Li

**Affiliations:** 1 Center for Molecular Medicine, Xiangya Hospital, Central South University, Changsha, China; 2 Hunan Key Laboratory of Molecular Radiation Oncology, Xiangya Hospital, Central South University, Changsha, China; 3 Department of Radiation Oncology, Tumor Hospital, Xinjiang Medical University, Urumqi, China; 4 Tumor Research Institute, Central South University, Changsha, China; 5 State Key Laboratory of Chemo/Biosensing and Chemometrics, Hunan University, Changsha, China; 6 School of Pharmaceutical Science, Central South University, Changsha, China; 7 Department of Pathology, Xiangya Hospital, Central South University, Changsha, China; 8 Department of Otorhinolaryngology, The Third Xiangya Hospital, Central South University, Changsha, China; 9 Department of Pharmacy, Xiangya Hospital, Central South University, Changsha, China; 10 Department of Infectious Disease, Xiangya Hospital, Central South University, Changsha, China

## Abstract

Paclitaxel is clinically used as a first-line chemotherapeutic regimen for several cancer types, including head and neck cancers. However, acquired drug resistance results in the failure of therapy, metastasis and relapse. The drug efflux mediated by ATP-binding cassette (ABC) transporters and the survival signals activated by forkhead box (FOX) molecules are critical in the development of paclitaxel drug resistance. Whether FOX molecules promote paclitaxel resistance through drug efflux remains unknown. In this study, we developed several types of paclitaxel-resistant (TR) nasopharyngeal carcinoma (NPC) cells. These TR NPC cells acquired cancer stem cell (CSC) phenotypes and underwent epithelial to mesenchymal transition (EMT), and developed multidrug resistance. TR cells exhibited stronger drug efflux than parental NPC cells, leading to the reduction of intracellular drug concentrations and drug insensitivity. After screening the gene expression of ABC transporters and FOX molecules, we found that FOXM1 and ABCC5 were consistently overexpressed in the TR NPC cells and in patient tumor tissues. Further studies demonstrated that FOXM1 regulated *abcc5* gene transcription by binding to the FHK consensus motifs at the promoter. The depletion of FOXM1 or ABCC5 with siRNA significantly blocked drug efflux and increased the intracellular concentrations of paclitaxel, thereby promoting paclitaxel-induced cell death. Siomycin A, a FOXM1 inhibitor, significantly enhanced *in vitro* cell killing by paclitaxel in drug-resistant NPC cells. This study is the first to identify the roles of FOXM1 in drug efflux and paclitaxel resistance by regulating the gene transcription of *abcc5*, one of the ABC transporters. Small molecular inhibitors of FOXM1 or ABCC5 have the potential to overcome paclitaxel chemoresistance in NPC patients.

Nasopharyngeal carcinoma (NPC) is a cancer type with a particularly high incidence in Southern China and Southeast Asian countries. Its annual incidence has been reported to be over 20/100000 in the Cantonese population in China.^1, 2^ NPC is initially sensitive to both radiotherapy and chemotherapy.^3^

Paclitaxel was approved by FDA to treat ovarian cancer in 1992^4^ and it has been commonly used to treat many cancer types, including breast, head and neck, lung and melanoma,^5^ as a first-line chemotherapeutic drug,. Paclitaxel, alone or in combined with cisplatin and/or 5-FU, has been tested as an adjuvant chemotherapeutic agent for advanced NPC patients in clinical trials.^6, 7, 8^ NPC patients are sensitive to chemotherapy at the beginning of treatment, but develop acquired resistance shortly after, resulting in therapy failure.^9, 10^ The molecular mechanisms of drug resistance are elusive. Previous studies have implicated many factors contributing to chemoresistance, including increased drug efflux or decreased drug influx, abnormal cellular metabolism, mutations of drug target genes, hyperactive DNA repair machines, deregulation of Bcl-2 apoptosis molecules, cell senescence, autophagy, tumor microenvironment and more.^11, 12^ Paclitaxel drug resistance has been closely associated with the modification of microtubulin in cancer cells, which may be induced by the point mutations, expression deregulation or posttranscriptional tubulin alterations.^12^ Hyperactive drug efflux and multidrug resistance (MDR) because of the overexpression of ATP-binding cassette (ABC) transporters, such as MRP1 and MDR1, are significant factors in the development of paclitaxel resistance.^9, 13^

The molecules of Forkhead box (FOX) family are reportedly involved in taxane-associated drug resistance.^14, 15^ Paclitaxel induces the expression of FOXO3a, and confers paclitaxel resistance by elevating BH3-only Bcl-2, or activating MAPK/JNK signaling.^16^ Among FOX molecules, FOXM1 has a critical role in the development of paclitaxel resistance.^14, 17, 18^ The overexpression of FOXM1 results in paclitaxel resistance, and FOXM1 depletion increases paclitaxel sensitivity.^17^ FOXM1 is a cell growth-specific transcription factor that regulates target genes controlling the G1/S and G2/M cell cycle transition.^19, 20^ The exact mechanisms of FOXM1 involvement in paclitaxel resistance remain undefined.

In this study, we developed several types of paclitaxel-resistant CNE2TR NPC cells by intermittently treating the cells with low doses of paclitaxel.^21^ CNE2TR cells exhibited resistance to paclitaxel, acquired the characteristics of cancer stem cells (CSCs) and underwent epithelial to mesenchymal transition (EMT). The paclitaxel-resistant cells developed MDR by decreasing the intracellular drug concentrations. FOXM1 and the ABC transporter ABCC5 were consistently overexpressed in paclitaxel-resistant NPC cells and tumor tissues. We explored whether FOXM1 contributed to paclitaxel resistance by regulating *abcc5* gene transcription and protein expression, thereby increased drug efflux. We also tested whether a FOXM1 inhibitor used as a chemosensitizer may restore paclitaxel sensitivity in cancer cells.

## Results

### NPC cells developed resistance to paclitaxel after long-term and intermittent exposure

**Figure 1:**
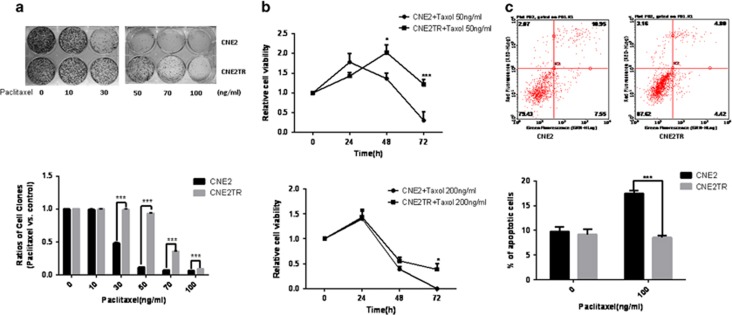
Assessment of paclitaxel-resistant NPC cell drug resistance. (**a**) Cell colony formation assay. Paclitaxel-resistant CNE2TR NPC cells and the parental CNE2 cells were treated with paclitaxel at stepwise concentrations for 48 h. One thousand cells were re-seeded in six-well plates, and cell clones were stained with crystal violet and analyzed 15 days after cell seeding. The cells were cleaved by 10% SDS, and cell viability was tested by spectrometer at a wavelength of OD570. (**b**) Cell viability assay (MTS). CNE2TR and CNE2 were treated with paclitaxel at 50 ng/ml or 200 ng/ml, and cell viability was tested by MTS assay 24, 48 and 72 h after treatment. The relative cell viability represents a ratio of paclitaxel treatment *versus* control. (**c**) Cell apoptosis detection assay. CNE2TR and CNE2 were treated with paclitaxel (100 ng/ml) for 24 h, cells were stained with Annex V/PI, and apoptotic cells were detected by flow cytometry. *P<0.05, ***P<0.001

We previously developed a paclitaxel-resistant cell line, CNE2TR, by intermittently exposing CNE2 cells to low doses of paclitaxel over a long period.^21^ The resistance of CNE2TR cells to paclitaxel was assessed by colony formation assay and apoptosis detection assay. Paclitaxel 30, 50, 70 and 100 ng/ml killed many more CNE2 cells than CNE2TR cells (Figure 1a). At the doses of 50 or 200 ng/ml, paclitaxel killed more CNE2 cells than CNE2TR cells 48 and 72 h after treatments (Figures 1b and c). At a dose of 100 ng/ml, paclitaxel induced more cell apoptosis in CNE2 cells than CNE2TR cells (Figure 1d). These data verified that CNE2TR cells are more resistant to paclitaxel than CNE2 cells.

### Paclitaxel-resistant NPC cells acquired CSC characteristics and underwent EMT

**Figure 2:**
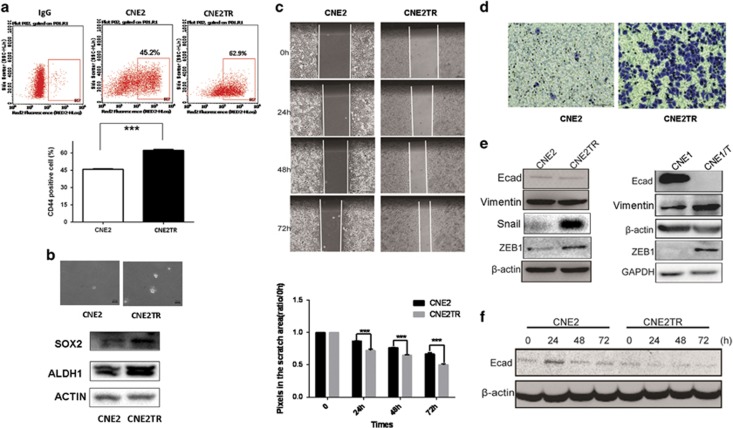
Paclitaxel-resistant cells increased as a sub-population of CD44+ CSCs and underwent EMT. (**a**) CSC sub-population. CNE2TR and CNE2 cells were labeled with fluorescent antibodies against CD44 (APC). CD44+ cells were detected by flow cytometry. (**b**) CNE2 and CNE2TR cells were seeded in soft agar for cell sphere formation. The protein levels of SOX2, SHH and ALDH1 were tested by western blot. (**c**) Cell migration assay. A confluent monolayer of CNE2TR and CNE2 cells was scratched. Displaced cells were moved, and the cell gaps were monitored at 24, 48 and 72 h after scratching. The cell gap was quantified by Image Pro Plus software and the data were presented as the cell gap distance. (**d**) Cell invasion (transwell) assay. CNE2TR/CNE2 cells were starved for 48 h, and re-plated on transwell plate inserts with serum-free media, whereas culture media with 10% FBS was placed in the bottom wells. Invasive cells on the membrane were stained by crystal violet 24 h after cell plating. (**e**) The expression of EMT-associated molecules was tested in CNE2TR/CNE2 and CNE1T/CNE1 cells. (**f**) The expression of E-cadherin proteins in NPC cells was monitored at 24, 48 and 72 h after paclitaxel treatment. ***P<0.001

First, we tested the proportion of CSCs among the CNE2TR and CNE2 cell populations. The proportion of CD44+ cells significantly increased in CNE2TR cells compared with CNE2 cells (62.9% *versus* 45.2%, Figure 2a). We further tested a smaller proportion of CD44^high^CD133^high^ cells. The percentage of CD44^high^CD133^high^ cells in the CNE2TR population markedly increased compared with CNE2 cells (1.57% *versus* 1%, Supplementary Figure S1). Cell spheres formed by CNE2 cells were fewer and smaller than those formed by CNE2TR cells, and the expression levels of SOX2, Sonic Hedgehog (SHH) and ALDH1, typical stem cell markers in CNE2TR cells, were much higher than in CNE2 cells (Figure 2b), indicating that the subgroup of paclitaxel-resistant CNE2TR cells acquired CSC characteristics. The *in vivo* tumorigenesis abilities of CNE2TR cells were much stronger than CNE2 cells.^21^ Cell migration and invasion capability were tested by wound-healing assay or transwell migration assay. At 24, 48 and 72 h after cell scratching, CNE2TR cells migrated much faster than CNE2 cells (Figure 2c), and cell invasion by CNE2TR was stronger than CNE2 cells (Figure 2d). Reportedly the phenotype transitions from epithelial to mesenchymal as cancer cells develop therapeutic resistance.^21, 22^ The expression levels of EMT-associated molecules were significantly altered in CNE2TR and CNE1/T cells (the drug resistance of this cell line had been tested; data not shown) compared with parental CNE2 or CNE1 cells. E-cadherin decreased, whereas Vimentin, Snail and ZEB1 markedly increased (Figure 2e). In the paclitaxel-resistant CNE2TR cells, paclitaxel (10 ng/ml) decreased the level of CNE2TR E-cadherin over time from 24 to 72 h (Figure 2f.). These data indicated that paclitaxel-induced EMT as the NPC cells developed resistance to paclitaxel treatment.

### Paclitaxel-resistant cells developed MDR

**Figure 3:**
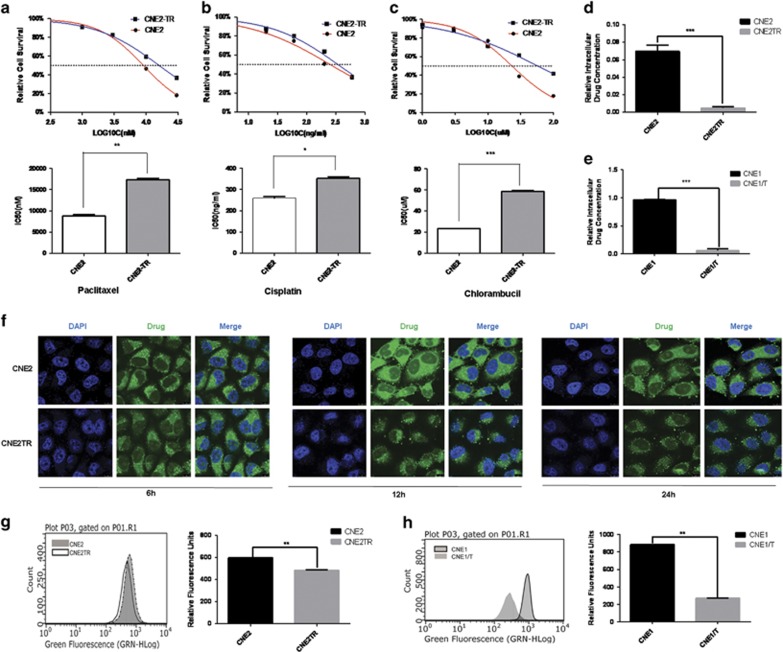
Paclitaxel-resistant NPC cells developed MDR and showed decreased intracellular drug concentrations. CNE2TR and CNE2 cells were treated with paclitaxel (**a**), cisplatin (**b**) or chlorambucil (**c**) at the doses as shown. MTS assays were used to test the cell viability 48 h after treatment with three repeats for each dose, and relative cell survival was calculated (treatment *versus* control) to compare the IC50 of drugs. Two pairs of NPC cells, CNE2TR/CNE2 (**d**) and CNE1TR/CNE1 (**e**) were used to test drug efflux. These cells were treated with 500 ng/ml paclitaxel for 2 h. The culture media were harvested for to test intracellular drug concentrations. The cells were completely washed, and then prepared by ultrasonic homogenization. The solution after spinning off cell debris was used to test intracellular drug concentrations. The drug concentrations were measured by UPLC-MS. (**f**) Intracellular drug concentrations were monitored by confocal microscopy. Fluorescent chlorambucil was synthesized by the conjunction with chlorambucil and the delocalized lipophilic cation probes rhodamine 123 and MKT-077. CNE2TR and CNE2 cells were treated with the fluorescent chlorambucil for 6, 12 and 24 h, and intracellular green fluorescence was monitored by confocal microscopy. (**g** and **h**) The strength of fluorescence was assessed by flow cytometry. CNE2TR/CNE2 or CNE1TR/CNE1 cells were treated with the rhodamine 123-labeled chlorambucil for 24 h, and fluorescence-positive cells were analyzed by flow cytometry. *P<0.05, **P<0.01, ***P<0.001

As paclitaxel promoted CNE2 cell EMT, we hypothesized that the paclitaxel-resistant CNE2 cells (CNE2TR) also were resistant to other chemotherapeutic drugs, that is, developed MDR. To verify this hypothesis, we tested the drug sensitivity of CNE2 and CNE2TR cells to the chemotherapeutic drugs cisplatin and chlorambucil in addition to paclitaxel. The sensitivities of CNE2TR and CNE2 cells to the three drugs were very consistent. Paclitaxel, cisplatin and chlorambucil induced more cell killing in CNE2 than CNE2TR. The IC50 of chemotherapeutic drugs in CNE2TR cells was much higher than CNE2 (Figures 3a–c). The results indicated that CNE2 cells developed MDR after long-term paclitaxel treatment. To test whether NPC cells developed MDR because of the selection of an innate paclitaxel-resistant subgroup, we tested the IC50 of CNE2TR-CD44+ cells and CNE2-CD44+ cells compared with parental CNE2TR cells and CNE2 cells. In both CNE2 and CNE2TR cells, the IC50 of CD44+ cells was much higher than the parental cells (CNE2: 474 *versus* 252.4 ng/ml; CNE2TR: 497.6 *versus* 342.3 ng/ml, Supplementary Figure S1b).

### Intracellular drug concentrations were significantly reduced in paclitaxel-resistant cells

As paclitaxel-resistant CNE2TR cells developed resistance to other chemotherapeutic drugs, we hypothesized that common pathways of MDR were probably activated in CNE2TR cells. We first tested the drug efflux of paclitaxel in CNE2TR cells compared with parental CNE2 cells. These cells were treated with paclitaxel, and cell culture media were harvested, for testing extracellular drug concentrations. Cells were prepared by ultrasonic homogenization after thorough washing, and intracellular drug concentrations were measured by UPLC-MS (Supplementary Figure S2). As shown in Figures 3d and e, intracellular drug concentrations apparently decreased in paclitaxel-resistant CNE2TR and CNE1/T cells compared with their parental cells. To track and compare drug efflux, we synthesized rhodamine 123-labeled chlorambucil by chemically integrating chlorambucil with a rhodamine 123 derivative probe (Supplementary Figure S3). The cells were treated with the rhodamine 123-labeled chlorambucil, and green fluorescence inside the cells was detected by confocal microscopy. At 6, 12 and 24 h, the fluorescence inside CNE2TR cells was noticeably weaker than CNE2 cells, indicating that the intracellular drug concentrations were remarkably lower than in CNE2 cells (Figure 3f). The results were consistently confirmed by flow cytometry. At 24 h after drug treatment, the strength of fluorescence in paclitaxel-resistant cells (CNE2TR or CNE1/T) significantly decreased compared with parental CNE2 or CNE1 cells (Figures 3g and h).

### ABC transporters, together with FOX molecules, were significantly overexpressed in paclitaxel-resistant NPC cells

**Figure 4:**
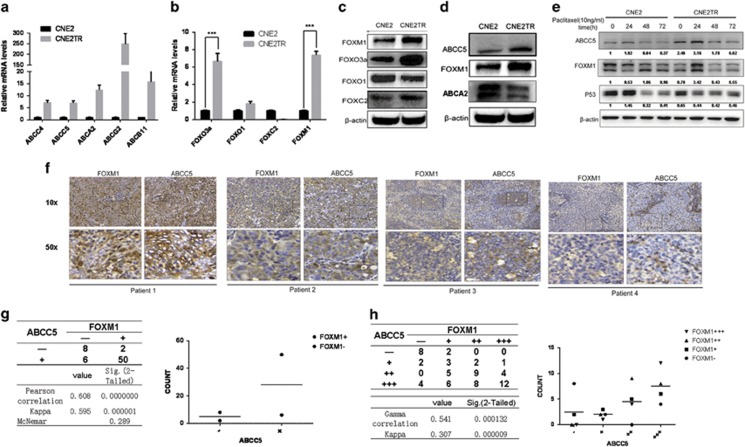
FOXM1 and ABCC5 were consistently upregulated in paclitaxel-resistant NPC cells and tumor tissues.(**a** and **b**) mRNA levels of ABC transporters and FOX genes in CNE2 and CNE2TR cells were analyzed by RT-PCR ***P<0.001. (**c**) Protein levels of FOX genes were analyzed by western blot. (**d**) ABCC5 and FOXM1 proteins were consistently overexpressed in CNE2TR cells compared with CNE2 cells. (**e**) The expression levels of FOXM1 and ABCC5 proteins in CNE2 and CNE2TR were detected at 24, 48 and 72 h after paclitaxel treatment (10 ng/ml). (**f**) Expression levels and localizations of FOXM1 and ABCC5 were detected by IHC in NPC tissues from 66 cases. (**g** and **h**) FOXM1 and ABCC5 expression levels were correlated by analysis using the Pearson correlation and kappa analysis methods, or Gamma correlation and kappa analysis methods. The correlation of FOXM1 and ABCC5 was regarded as low when the correlation factor was ≤0.4, intermediate when it was between 0.4 and 0.75, and high when it was over or equal to 0.75

As drug efflux was significantly increased in paclitaxel-resistant NPC cells (Figure 3), we deduced that increased drug efflux may be driven by the elevated expression of ABC transporters. We screened the expression levels of 16 ABC transporter family members in both CNE2TR and CNE2 cells (Supplementary Figure S4a and b). Of these ABC transporter molecules, 6 out of 16 were significantly upregulated in CNE2TR cells compared with CNE2 cells (Figure 4a). Such FOX family molecules as FOXM1, FOXO3a, FOXO1 and FOXC2 have been reported to have significant roles in chemoresistance.^15^ We compared their expression levels in CNE2TR and CNE2 cells. Higher expression levels of FOXO3a, FOXM1 and FOXC2 were detected in CNE2TR than CNE2 at both mRNA and protein levels (Figures 4b and c).

We further analyzed correlations between FOX molecules and ABC transporters by testing the response of ABC transporters to the depletion of individual FOX molecules. FOXM1 depletion significantly lowered the mRNA levels of ABCA2 and ABCC5, a positive correlation (Supplementary Figure S5a). The protein levels of FOXM1 and ABCC5 were consistently elevated in CNE2TR cells compared with the parental CNE2 cells, but ABCA2 protein elevation was not detected in CNE2TR cells (Figure 4d). In addition, we detected elevated FOXM1 and ABCC5 mRNA levels in paclitaxel-resistant ovarian cancer SKOV3R cells compared with parental SKOV3 cells (Supplementary Figure S6). To test whether the increased FOXM1 and ABCC5 resulted from paclitaxel treatment, we monitored the protein expression levels of FOXM1 and ABCC5 in CNE2 and CNE2TR cells at 24, 48 and 72 h after paclitaxel treatment. p53 protein was elevated in CNE2 cells but decreased in CNE2TR at 24 h (Figure 4e). Consistent protein kinetics of FOXM1 and ABCC5 were observed in both cell lines. In CNE2TR cells, the levels of FOXM1 and ABCC5 significantly increased at 24 h upon paclitaxel treatment, whereas FOXM1 and ABCC5 expression did not increase at 24 h, but decreased thereafter in response to paclitaxel treatment in CNE2 cells. Intriguingly, the expression of ABCC5 followed similar kinetics as FOXM1 upon paclitaxel treatment in both cell lines, indicating the close correlation between FOXM1 and ABCC5 (Figure 4e).

### FOXM1 and ABCC5 were consistently expressed in NPC tumor tissues

We tested the correlation of FOXM1 and ABCC5 expression in 66 squamous tumor tissues from NPC patients. FOXM1 and ABCC5 proteins in the tumor tissues were stained by immunohistochemistry. FOXM1 protein was detected in both cytoplasm and nuclei, whereas ABCC5 protein was detected in the cytoplasm only (Figure 4f). FOXM1 and ABCC5 were consistently expressed in these same tumor tissues, although the clinical history of patients and their sensitivity to chemotherapeutic agents were unavailable,. ABCC5 expression was low when FOXM1 expression was low in the same tumor tissue and the higher FOXM1 expression, the stronger was ABCC5 expression in the same tumor tissues (Figure 4f). Protein expression results were evaluated by two independent senior pathologists. These tumor tissues were classified into two groups (positive + or negative –), and the positive tissues were further graded into four levels according to the degrees of FOXM1 or ABCC5 expression (negative, +, ++, +++). The results of statistical analysis demonstrated very strong correlations between FOXM1 and ABCC5 protein expression in both groups (Pearson correlation 0.608, *P*=0.000000, kappa 0.595, *P*=0.000001) and all four levels (Gamma correlation 0.541*, P*=0.000132, kappa 0.307, *P*=0.000009) (Figures 4g and h).

### FOXM1 regulates *abcc5* gene transcription

**Figure 5:**
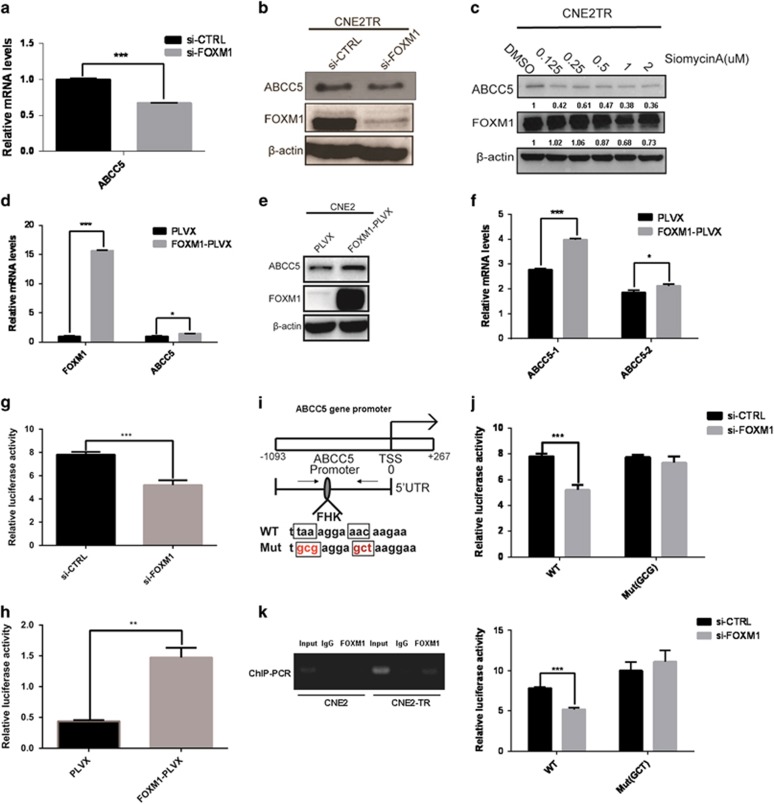
FOXM1 regulates *abcc5* gene transcription. (**a** and **b**) The depletion of FOXM1 with siRNA in paclitaxel-resistant CNE2TR cells decreased the expression of ABCC5 at mRNA and protein levels. (**c**) The depletion of FOXM1 with a small molecular inhibitor, siomycin A, in CNE2TR cells decreased the expression of ABCC5 in a dose-dependent manner. (**d** and **e**) The elevation of FOXM1 in CNE2 cells elevated the mRNA levels of *abcc5* gene and the protein levels of ABCC5. (**f**) The overexpression of FOXM1 elevated the levels of two mRNA splicing variants of the *abcc5* gene, *abcc5-1* and *abcc5-2*.(**g**) The depletion of FOXM1 with siRNA in CNE2TR cells decreased *abcc5* gene promoter activity. (**h**) The overexpression of FOXM1 in HEK293T cells elevated *abcc5* gene promoter activity. (**i**) FOXM1 binding to *abcc5* gene promoter was impaired when the core sequences of the FHK consensus binding motif were mutated (the TAA was mutated to GCG, or the AAC was mutated to GCT). (**j**) The luciferase activity of *abcc5* gene promoter was tested when the CNE2TR cells were co-transfected with *abcc5* gene promoter and FOXM1 siRNA. (**k**) Primers spanning the FHK binding motif were designed, and the binding of FOXM1 protein to *abcc5* gene promoter was detected by ChIP-PCR. *P<0.05, **P<0.01, ***P<0.001

FOXM1 is a cell growth-specific transcription factor that regulates the transcription of cell cycle regulatory genes, which are closely associated with G1/S and G2/M transitions.^19^ Recent whole-genome studies suggested that *abcc5* may be a target gene regulated by FOXM1, as ChIP-seq data showed a very strong binding peak in the enhancer/promoter areas of the *abcc5* gene.^23^ We tested whether FOXM1 regulates the transcription of *abcc5* gene. In CNE2TR cells, the mRNA and protein levels of ABCC5 obviously decreased when FOXM1 was depleted by siRNA (Figures 5a and b). Siomycin A, a small molecular inhibitor of FOXM1, induced the degradation of FOXM1 protein in a dose-dependent manner. With the degradation of FOXM1 proteins, the ABCC5 protein levels correspondingly decreased dose dependently (Figure 5c). We tested ABCC5 expression at the mRNA and protein levels when FOXM1 was elevated in CNE2 cells by complementary DNA (cDNA) transient transfection. With the elevation of FOXM1, the mRNA and protein levels of ABCC5 remarkably increased, although the extent of the elevation was not as significant as FOXM1 (Figures 5d and e). As the *Abcc5* gene has two splicing variants, *abcc5-1* and *abcc5-2*, we further tested whether the two splicing variants of *abcc5* responded differentially to FOXM1 elevation. Indeed, *abcc5-1* responded to FOXM1 elevation more significantly than *abcc5-2* (Figure 5f).

To clarify the mechanisms by which FOXM1 regulates *abcc5* gene transcription, we tested the promoter activity of *abcc5* gene using a luciferase reporter assay. A 1365-bp promoter of *abcc5* gene was used for the test. Consistent with the response of ABCC5 to FOXM1 changes at the mRNA and protein levels, the promoter activity of *abcc5* gene significantly decreased when FOXM1 was knocked down with siRNA in CNE2TR cells (Figure 5g). In reverse, with the elevation of FOXM1 proteins in CNE2 cells, the promoter activity of *abcc5* gene significantly increased (Figure 5h). Furthermore, we verified whether FOXM1 regulates *abcc5* gene transcription by binding to the FHK consensus motif at the *abcc5* gene promoter. Fox proteins bind the core consensus sequence (A/C)AA(C/T). The core sequence of FHK consensus binding motif taaAGGAaac at the promoter of *abcc5* gene was mutated to gcgAGGAaac or taaAGGAgct (Figure 5i), and promoter activity was tested after FOXM1 siRNA interference. The *abcc5* gene promoter with the mutations of FHK motif lost the response to FOXM1 knockdown, whereas the activity of wild-type *abcc5* gene promoter decreased with the knockdown of FOXM1 (Figure 5j). The binding of FOXM1 to the FHK consensus motif at the *abcc5* gene promoter was validated by ChIP-PCR. In CNE2 cells, the binding is weak, whereas the binding was stronger in paclitaxel-resistant CNE2TR cells (Figure 5k). These data indicated that FOXM1 regulates *abcc5* gene transcription by binding to the FHK motif in the promoter, and the stronger FOXM1 binding to *abcc5* gene promoter in CNE2TR cells probably resulted in elevated ABCC5 levels, which led to paclitaxel resistance.

### The depletion of FOXM1 or ABCC5 increased the intracellular concentration of chemotherapeutic agents, and sensitized the resistant cells to paclitaxel treatment

**Figure 6:**
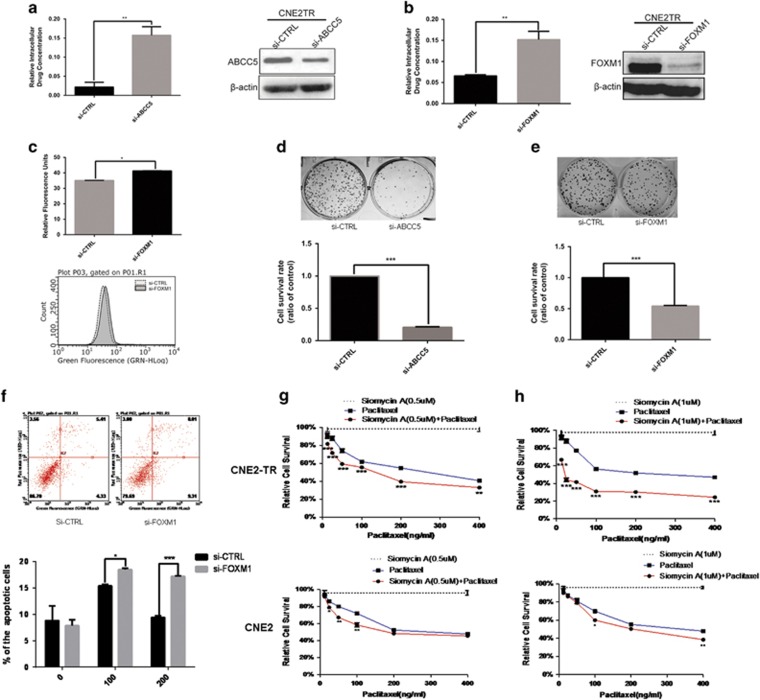
FOXM1 or ABCC5 depletion increased the intracellular concentrations and sensitized the paclitaxel-resistant cells to paclitaxel treatment. (**a**) ABCC5 was knocked down by siRNA in CNE2TR cells, with the gene knockdown efficiency confirmed by western blot. (**b**) FOXM1 was knocked down by siRNA in CNE2TR cells, and the gene knockdown efficiency was confirmed by western blot. The cells were treated with 500 ng/ml paclitaxel for 2 h. The culture media were harvested to test intracellular drug concentrations. Cells were completely washed, and the cells were prepared by ultrasonic homogenization. The solution after spinning the cell debris was used to test of intracellular drug concentrations. The drug concentrations were measured by UPLC-MS. (**c**) FOXM1 was depleted by siRNA in CNE2TR cells, and the gene knockdown efficiency was confirmed by western blot. The cells were treated with fluorescent chlorambucil for 24 h, and cell fluorescence was analyzed by flow cytometry. (**d** and **e**) FOXM1 or ABCC5 was knocked down by siRNA in CNE2TR cells, with the efficiency of gene knockdown validated by western blot. The cells were treated with paclitaxel (100 ng/ml) for 48 h and re-seeded (1000 cells per treatment) in six-well plates for 15 days, when cell colonies were stained with crystal violet. The cells were cleaved by 10% SDS, and cell viability was tested at the OD570 wavelength by spectrometer (five repeats per sample). (**f**) FOXM1 was knocked down by siRNA in CNE2TR cells, and cells were treated with paclitaxel (100 or 200 ng/ml) for 24 h. The cells were stained with Annex V/PI, and apoptotic cells were detected by flow cytometry. (**g** and **h**) CNE2 or CNE2TR cells were seeded in 24-well plates and treated with siomycin A or/and paclitaxel at the doses shown for 48 h. Cell viability was tested by MTS assay. (**g**) Siomycin A (0.5 *μ*M) alone or in combination with paclitaxel. (**h**) Siomycin A (1 *μ*M) alone or in combination with paclitaxel. *P<0.05, **P<0.01, ***P<0.001

We monitored the alterations of intracellular drug concentrations when ABCC5 or FOXM1 was depleted with siRNAs. Cells with FOXM1 or ABCC5 depletion were treated with paclitaxel, and the intracellular concentrations were measured by UPLC-MS. With the knockdown of ABCC5 or FOXM1, intracellular drug concentrations significantly increased (Figures 6a and b). We also tested the intracellular drug concentrations of rhodamine 123-labeled chlorambucil by analyzing the fluorescence of cells by flow cytometry when ABCC5 or FOXM1 was depleted with siRNAs. Consistently, the intracellular drug concentrations significantly increased with FOXM1 knockdown (Figure 6c).

As FOXM1 and ABCC5 are critical to the drug efflux of chemotherapeutic agents, we tested whether the depletion of FOXM1 or ABCC5 would promote cell killing by paclitaxel. As shown in Figures 6d and e, CNE2TR cells became more sensitive to paclitaxel treatment when ABCC5 or FOXM1 were knocked down with siRNAs. The number of cell colonies significantly decreased in ABCC5 or FOXM1 knockdown cells compared with control cells. Paclitaxel-induced cell apoptosis significantly increased with the knockdown of FOXM1 at doses of 100 and 200 ng/ml (Figure 6f). Siomycin A, a small molecular inhibitor of FOXM1, alone killed CNE2TR cells when the doses were over 1* μ*M (Supplementary Figure S7a), and induced apoptosis in CNE2TR cells when the doses were over 1 *μ*M in a dose-dependent manner (Supplementary Figure S7b). Finally, siomycin A at the dose of 0.5 and 1 *μ*M sensitized CNE2TR cells to paclitaxel at doses of 12.5, 25, 50, 100, 200 and 400 ng/ml, but the effect was not as significant in CNE2 cells (Figures 6g and h). These data demonstrated the potential of siomycin A to sensitize chemoresistant cancer cells to paclitaxel.

## Discussion

In this study, paclitaxel-resistant NPC cells developed MDR when they had been treated with long-term low doses of paclitaxel. More cells acquired CSC and mesenchymal-like cell characteristics after treatment. Crosstalk between EMT and CSC signaling pathways has been reported. Such molecules as Wnt, SHH and Notch have been activated when the cancer cells developed chemoresistance, and are critical for CSC self-renewal and maintenance.^24^ These CSC-associated molecules contribute to cancer cell EMT^25, 26, 27^ and drug resistance^28, 29^ as well.

Paclitaxel-resistant CNE2TR NPC cells pumped more drugs out of the cells, resulting in lower drug intracellular concentrations. Drug efflux is a critical mechanism by which cancer cells develop chemoresistance. Cancer cells obtain stronger drug efflux ability as an adaptation to chemotherapy. Drug efflux or MDR is controlled by the elevated expression of ABC transporters, which consist of 7 subfamilies and 49 molecules in humans.^30^ These transporters function as drug efflux pumps to transport various molecules across extra- and intracellular membranes. Drug resistance is induced by the hyperactive extrusion of anticancer drugs. Each molecule of the ABC transporters has been identified to specifically control the drug efflux of one or several agents depending on their expression levels in different tissues.^30^ In breast samples, the overexpression of ABC transporters such as ABCA1, ABCA12, ABCB1 and ABCB6 have been reported to control the drug efflux of paclitaxel.^30^ ABCC5 is one of the critical ABC transporter molecules involved in paclitaxel drug resistance.^31, 32, 33^ ABCC5 contributes to paclitaxel resistance in the NPC cells.^33^ In this study, ABCC5 was overexpressed in paclitaxel-resistant NPC cells, and the expression level was positively correlated with drug efflux and drug resistance. The depletion of ABCC5 with siRNA significantly decreased the drug efflux, thereby increasing the intracellular concentrations of paclitaxel to overcome paclitaxel drug resistance.

Promoters of ABC transporters reportedly contain several binding sites for EMT-associated transcription factors such as Twist, Snail and FOXC2. Overexpression of these transcription factors increases promoter activity and the expression of ABC transporters in breast cancer cells.^34^ *abcc5* gene transcription is regulated by differential cooperation of NRF2 and HER2.^35^ In *abcc5* gene promoter, a number of consensus binding motifs for transcription factors have been identified. However, which transcription factors bind to the promoter to regulate *abcc5* gene transcription depends on the particular cell lines and environments. FOX transcription factor superfamily members such as FOXC2 and FOXM1 have been reported to promote drug resistance.^21, 36^ FOXM1 is a cell growth-specific transcription factor that regulates the cell cycle regulatory genes involved in the G1/S and G2/M checkpoints.^20^ FOXM1 contributes to paclitaxel resistance by regulating the target genes associated with cell cycle and DNA repair,^15^ the tubulin destabilizing protein Stathmin^17^ and microtubulin-associated kinesin KIF20A^37^ and KIF-2C.^18^ Interference with FOXM1 has been reported to re-sensitize cancer cells to chemotherapeutic drugs.^17^ ChIP-seq data have demonstrated that FOXM1 directly binds to the enhancer/promoter area of *abcc5* gene in the breast cancer cell line MCF-7.^23, 38^ By screening 16 members of the ABC transporter family and 4 chemoresistance-associated FOX molecules, we found a very high correlation between FOXM1 and ABCC5 in the cells and the tumor tissues of NPC patients. Ectopic overexpression or knockdown of FOXM1 consistently increased or decreased *abcc5* gene expression. Furthermore, FOXM1 regulates the *abcc5* gene by binding to the FHK consensus binding motifs at the *abcc5* gene promoter. The binding of FOXM1 is much stronger in CNE2TR than CNE2 cells. The results indicated that FOXM1 bound to the promoter to regulate *abcc5* gene transcription. The elevation of ABCC5 resulted in paclitaxel resistance.

Certainly the molecular mechanisms by which paclitaxel resistance develops include many other complicated pathways. The results of this study only provide evidence for the involvement of the FOXM1–ABCC5 axis in the development of paclitaxel resistance, but did not address the interactions of these signals with other pathways that have been reported in different models and cancer types.

Our study found that ABCC5, an ABC transporter family molecule, is involved in NPC paclitaxel resistance and that *abcc5* gene transcription is regulated by FOXM1. FOXM1 has been reported to be involved in chemoresistance, but our study is the first to report that FOXM1 induced drug resistance by regulating *abcc5* gene transcription and drug efflux. Our findings suggest a therapeutic protocol for clinical trials simultaneously targeting FOXM1 and ABCC5 to overcome paclitaxel drug resistance in NPC patients.

## Materials and Methods

### Cell culture

The CNE2 and CNE1 NPC cell lines were obtained from the Cancer Research Institute of Central South University, Changsha, China. The paclitaxel-resistant cell sublines CNE2TR, CNE1/T and SKOV3R were established by intermittently exposing the parental cells to gradually increasing concentrations of paclitaxel.^33^ These cell lines were cultured in RPMI 1640 medium (Hyclone, GE Healthcare Life Science, Logan, UT, USA) containing 10% fetal bovine serum with 1% penicillin/streptomycin (Invitrogen, Thermo Fisher Scientific, Waltham, MA, USA) at 37 °C in a 5% CO_2_ incubator. Paclitaxel-resistant cells were maintained in culture medium supplemented with 1 nM paclitaxel (Bristol-MyersSquibb, Princeton, NJ, USA).

### Plasmids and stable cell lines

Human FOXM1 cDNA was synthesized and then cloned into the pLV-EF1*α*-MCS-IRES-Bsd lentiviral vector (Biosettia, San Diego, CA, USA). The FOXM1-expressing or control lentivirus was produced by co-transferring the plasmids with VSV-G, Rev, and Gag-Pol to HEK-293 T cells. FOXM1-overexpressing CNE2 cells were generated by infecting CNE2 cells with lentivirus for 48 h, followed by selection using 8 *μ*g/ml BSD for 2 weeks.

The following primers were designed to synthesize FOXM1 shRNA: upper: 5'-GATCCGCTCTTCTCCCTCAGATATATTCAAGAGATATATCTGAGGGAGAAGAGTTTTTTG-3'; lower: 5'-AATTCAAAAAACTCTTCTCCCTCAGATATATCTCTTGAATATATCTGAGGGAGAAGAGCG-3'). The shRNA was synthesized and cloned into Lenti-X shRNA Expression Systems (Clontech, Mountain View, CA, USA). The FOXM1 knockdown or control lentivirus was produced by co-transferring the plasmids with VSV-G, Rev, and Gag-Pol to HEK-293 T cells. FOXM1 stable knockdown CNE2TR cells were generated by lentivirus infection for 48 h, followed by selection using 2 *μ*g/ml puromycin for 2 weeks.

### MTS assay

Cells were cultured overnight (5000 per well) in 96-well flat-bottomed microtiter plates exposed to paclitaxel at 50 or 200 ng/ml. At individual time points of 0, 24, 48 and 72 h, 20 *μ*l of MTS solution [3-(4,5-dimethylthiazol-2-yl)-5-(3-carboxymethoxyphenyl) -2-(4-sulfophenyl)-2Htetrazolium] (Promega, Madison, WI, USA) was added to each well, and cultured at 37 °C in 5% CO_2_ for 4 h. Absorbance at 490 nm was measured by spectrometer. Each treatment was performed in quintuplicate. The relative viability of the cancer cells was calculated.

### Colony-forming assay

CNE2 or CNE2TR cells were treated with stepwise concentrations of paclitaxel for 48 h. To test the sensitivity of CNE2TR cells to paclitaxel when FOXM1 or ABCC5 was knocked down, we first transfected FOXM1 or ABCC5 siRNA to CNE2TR cells for 24 h, followed by paclitaxel treatment of (100 ng/ml) for an additional 48 h. CNE2TR cells were treated with stepwise concentrations of siomycin A (Calbiochem, San Diego, CA, USA) for 48 h, and DMSO was used as a negative control. The cells were re-seeded in six-well plates (1000 cells per well) after treatments as above, and then cultured for 15 days for colony formation. Each treatment was performed in triplicate. The cell colonies were fixed in 3.7% paraformaldehyde and stained by 0.05% crystal violet solution. The dishes were photographed after staining. The cells were cleaved by 10% SDS, and the cell survival ratio was assessed by measuring absorbance at 570 nm.

### RNA extraction and real-time quantitative PCR

The total RNA was isolated by the RNAiso Plus method following the manufacturer's protocol. The dissolved RNA sample was measured on a spectrophotometer to determine concentration and quality before its conversion to cDNA. Quantitative real-time PCR was then performed using the CFX96TM Real-Time PCR Detection System (Bio-Rad, Hercules, CA, USA) according to the manufacturer's instructions. Values were expressed as fold changes compared with the corresponding values for the control using the 2 –ΔΔCt method.

### Western blot analysis and antibodies

The cells were harvested with RIPA buffer. The protein samples (30–50 *μ*g) were separated by SDS-PAGE and transferred onto polyvinylidene difluoride (PVDF) membranes (Millipore, Bedford, MA, USA). The blots were blocked for 1 h, and then incubated with the primary antibodies at 4 °C overnight. The blots were incubated for 1 h with horseradish peroxidase-conjugated secondary antibodies (Santa Cruz Biotechnology, Dallas, TX, USA). The protein was visualized with a Pierce ECL Western Blotting Substrate detection system (Thermo Fisher Scientific, Bremen, Germany). *β*-Actin was used as the loading control. The primary antibodies used in this study included anti-FOXM1, anti-FOXO3a, anti-p53, anti-ABCA2, anti-SOX2, anti-ALDH1, anti-GAPDH and anti-*β*-actin purchased from Santa Cruz (Santa Cruz, CA, USA); anti-FOXO1, anti-FOXC2 and anti-ABCC5 purchased from Abcam (Shanghai, China); and anti-E-cadherin, anti-vimentin, anti-Snail, anti-ZEB-1, anti-p38, anti-p44/42, anti-AKT, anti-AKT473 and anti-AKT308 purchased from Cell Signaling Technology (CST, Danvers, MA, USA).

### Gene knockdown with siRNAs

For gene knockdown, cells were transiently transfected with siRNA or siRNA SMARTpool reagents purchased from GenePharma (Shanghai,China) or Thermo Scientific Dharmacon (Lafayette, CO, USA) using DharmaFECT Transfection Reagents (Thermo Fisher Scientific, Bremen, Germany) according to the manufacturer's instructions.

The sequences of siRNAs used in this study were: siRNA FOXM1 (sense: 5′-CUCUUCUCCCUCAGAUAUATT-3′), siRNA FOXO1 (sense: 5′-GCAGUAGAUACAGAUUGUATT-3′), siRNA FOXO3a (sense:5′-GCAUUAGGCAUAUAAAUGUTT-3′), siRNA FOXC2 (sense:5′-AGAAGGACGUGUCCAAGGATT-3′), siRNA ABCC5 (M-007614-02-0005, Thermo Scientific Dharmacon) non-target negative control siRNA (5′-UUCUCCGAACGUGUCACGUTT-3′), which has been confirmed to have minimal targeting of known genes.

### Flow cytometric analysis

Cells were suspended in PBS and incubated with anti-CD44 (APC-conjugated, BD PharMingen, San Jose, CA, USA) and anti-CD133 (PE-conjugated, Miltenyi Biotec, San Diego, CA, USA). Positive-staining cells were analyzed by Flow cytometry (Millipore, Temecula, CA, USA). To evaluate cell apoptosis, cells were collected and washed twice with PBS while spinning at 1000 r.p.m. for 10 min. Cell pellets were resuspended in a FITC-labeled Annexin V and propidium iodide (PI) staining solution (Annexin V-FITC Apoptosis Detection Kit, KeyGEN BioTECH, Jiangsu, China) and incubated for 15 min at room temperature. The apoptotic cells were then analyzed by flow cytometry (Millipore).

### Luciferase reporter assay

The human *abcc5* gene promoter was cloned by PCR, and subcloned into pGL3-TATA vector. A FHK consensus binding motif was mutated using a Quickchange site-directed mutagenesis kit (Thermo Fisher Scientific). CNE2TR cells were co-transfected with the gene promoter (WT or MUT), siFOXM1 RNA and pRL-SV40 (Promega) using the TransIT-X2 Dynamic Delivery System (Mirus, Madison, WI, USA). The firefly/Renilla luciferase activities were detected by the Dual-Glo Luciferase reporter assay system (Promega) according to the manufacturer's manual 48 h after transfection. Luminescence was then measured using the Perkin Elmer (EnSpire 2300) Multilabel Reader (PerkinElmer, Turku, Finland).

### Wound-healing and transwell migration assays

CNE2 and CNE2TR cells were plated (2 × 10^5^ cells per well) in six-well plates. A confluent monolayer of CNE2TR and CNE2 cells was scratched using a sterile 200 *μ*l plastic pipette tip. Displaced cells were moved with three washes, and the cell gaps were monitored at 24, 48 and 72 h after scratching. The cell gap was quantified by Image Pro Plus software (Media Cybernetics, Silver Spring, MD, USA) and data are presented as cell gap distance. CNE2 and CNE2TR cells were cultured in serum-free RPMI 1640 medium for 48 h, the cells were re-plated on transwell plate inserts with serum-free media, and normal culture media with 10% FBS was put in the bottom wells. The invasive cells on the membrane were stained by Violet Crystal 24 h after cell plating. Each treatment was performed in triplicate.

### Intracellular drug concentrations

#### Sample preparation

CNE2 and CNE2TR cells (2 × 10^6^ each well in six-well plate) were treated with paclitaxel (standard sample, 500 ng/ml) for 2 h. The cell culture media were harvested to test extracellular drug concentrations. The cells were prepared by ultrasonic homogenization after thorough washing, and 500 *μ*l supernatants were mixed with methanol. The samples were dried in a vacuum-dryer and re-dissolved in 200 *μ*l HPLC-grade methanol. The drugs were measured by liquid chromatography (LC)/electrospray ionization (ESI)-tandem MS using the UltiMate 3000 UPLC system online coupled to an linear trap quadrupole-Orbitrap Velos Pro mass spectrometer (Thermo Fisher Scientifc).

#### LC-tandem mass spectrometric analysis

The analytes (10 *μ*l) were separated by a C18 RP LC column (150 mm × 2.1 mm, 3 *μ*m, Thermo Fisher Scientifc) and eluted with a binary system consisting of solvent A (0.1% formic acid in aqueous phase) and solvent B (0.1% formic acid in methanol) at 1:1 with a flow rate of 0.3 *μ*l/min. An ESI source was applied and operated in the positive ion mode. The capillary voltage was 3.5 kV. For MS conditions, nitrogen (N_2_) was used as the sheath gas at a flow rate of 35 l/min. The aux gas (N_2_) flow rate was 8 l/min. The molecular weight of paclitaxel is 876. The method was linear in stepwise concentrations of 110, 130, 220, 440, 880 and 1760 ng/ml with the coefficient correlation of 0.999. The procedure was repeated in three biological replicates.

### Flow cytometry and confocal microscopy to test the intracellular concentrations of fluorescent drugs

Rhodamine 123-labeled chlorambucil was generated by chemical synthesis. CNE2 and CNE2TR cells were treated with rhodamine 123-labeled chlorambucil (10 mM) for 24 h. The rhodamine 123-positive cells were analyzed by flow cytometry. The cells were seeded and fixed on a glass slide, and the nuclei were stained with DAPI. The green fluorescence of cells inside the cytoplasm or nuclei was observed by confocal laser scanning microscopy (CLSM, Leica, Heidelberg, Germany).

### Immunohistochemistry

Paraffin sections of NPC tumor tissues were obtained from the Department of Pathology, Xiangya Hospital, Central South University. The slides were incubated with primary antibody against FOXM1 (Santa Cruz) and ABCC5 (Origene, Rockville, MD, USA), respectively. For negative control, isotype-matched antibodies were applied. FOXM1 staining was detected in both nuclear and cytoplasmic compartments, whereas ABCC5 staining was detected in both cytoplasm and membrane. The IHC results were judged from 5 to 10 random fields (× 400) by two independent senior pathologists. The degree of staining was classified into four stages (– negative, + slight yellow, ++ brown, +++ dark brown or tan). The ratio of positive cells was divided into three stages (+ <25%, ++ 25 to 49%, +++ ≥50%). The overall standard including staining degree and ratio of positive cells: +percent of positive cells × 1, ++ percent of positive cells × 2 and +++ percent of positive cells × 3. The overall results: +<1, ++1 to 1.5, +++>1.5.

### Statistics

All *in vitro* experiments were done either in triplicate or in quintuplicate. The analyses were conducted with Graphpad 6.0 software (GraphPad Software, Inc., La Jolla, CA, USA). The results are described as the mean±S.D. with analysis by one-way ANOVA or two-way ANOVA, and protein and mRNA expression levels were compared by two-sided Student's *t*-tests. The correlation between FOXM1 and ABCC5 expression in tumor tissues was assessed by Pearson correlation or Gamma correlation, and kappa analysis. A *P-*value of <0.05 was considered to be statistically significant (*P<0.05, **P<0.01, ***P<0.001). The correlation of FOXM1 and ABCC5 was regarded as low when the correlation factor was less or equal to 0.4, intermediate when it was between 0.4 and 0,75, and high when it was over or equal to 0.75.
