# Supplementary material for: The FOXM1–ABCC5 axis contributes to paclitaxel resistance in nasopharyngeal carcinoma cells
Source: Cell Death Dis. 2017 Mar 9;8(3):e2659–. doi: 10.1038/cddis.2017.53 (PMC5386553; doi:10.1038/cddis.2017.53)
Supplement: Supplementary Figures legend [file cddis201753x1.doc]

**Supplemental Figures**

**Figure S1.** The survival of paclitaxel-resistant cells increased the sub-population of CD133^high^CD44^high^ cancer stem cells (CSC). **(a)** The cancer stem cell sub-population. CNE2TR and CNE2 cells were labeled with fluorescent antibodies against CD133(PE) and CD44(APC). CD133^high^CD44^high^ cells were detected by flow cytometry. **(b)** CD44+ CSC cells were more resistant to paclitaxel than parental CNE2 and CNE2TR cells. The CD44+ CSC cells were isolated from CNE2 or CNETR cells and the IC50 of CNE2-CD44+ and CNE2TR-CD44+ cells were tested and compared with CNE2 and CNE2TR cells. **(c)** Two siRNAs targeting two different sequences of FOXM1 were designed. The knockdown efficiency of siRNAs were tested. The siRNA-1 was used in all following experiments.

**Figure S2. Measurement and calculation of intracellular paclitaxel concentration by Mass Spectrometer.**

Samples were prepared and measured by HPLC-MS-MS according to the protocol described in Materials and Methods. The procedure was repeated in three biological replicates. **(a)** and **(b)** The peak of paclitaxel was first identified by LC-MS-MS, and **(c)**　the area under the parabola was calculated to represent the amount of paclitaxel according to a formula: y=2.0×10^7^x+1.0×10^6^ (y: the area under the parabola; x: drug concentrations). The drug concentration was calculated as x(ug/ml)=(y-1000000)/20000000. **(d)** Linear method in stepwise concentrations of 110, 130, 220, 440, 880 and 1760 ng/mL with a coefficient correlation of 0.999.

**Figure S3. The molecular structure of the synthetic fluorescent chlorambucil (FC).**

FC is a conjugate of chlorambucil (blue, C) and the delocalized lipophilic cations (red, F). FC emits green fluorescence with an emission peak wavelength of 538 nm under 488 nm laser excitation, since the F part has green fluorescence as delocalized lipophilic cations probes of Rhodamine 123 and MKT-077. FC was used to assess drug efflux and influx.

**Figure S4. Gene expression of ABC transporters in CNE2 and CNE2TR cells.**

The total RNA of CNE2 and CNE2TR cells was extracted and converted to complementary DNA (cDNA). Quantitative real-time PCR was then performed. The mRNA levels of sixteen ABC transporter members were tested. **(a)** Genes with a CQ value over 30 were excluded due to low expression levels. **(b)** The relative mRNA levels were compared in CNE2 and CNE2TR cells, and genes with over 5-fold amplification were selected for the further analysis.

**Figure S5. Regulation of FOX proteins in ABC transporter gene transcription.**

Seven ABC transporter molecules were selected to test whether FOX proteins positively regulate the gene transcription of ABC transporters. CNE2TR cells were transfected with siRNAs for FOXM1 (a), FOXO3a (b), FOXC2(c) and FOXO1 (d). A non-target siRNA was used as a negative control. The total RNA of cells was isolated and converted to complementary DNA (cDNA). Quantitative real-time PCR was then performed to test the changes of ABC transporters when FOX genes were knocked down.

**Figure S6. FOXM1 and ABCC5 are overexpressed in other paclitaxel-resistant cells besides SKOV3/T cells.**

Pairs of SKOV3/SKOV3/T paclitaxel-resistant cells were used to test the mRNA levels of *foxm1* or/and *abcc5*. The total RNA of cells was isolated and converted to complementary DNA (cDNA). Quantitative real-time PCR was performed. (a) *foxm1* in SKOV3 and SKOV3/T cells; and (b) *abcc5* in SKOV3 and SKOV3/T cells.

**Figure S7. The small molecular inhibitor of FOXM1 siomycin A has the potential to overcome NPC cell paclitaxel-resistance.**

(a) CNE2TR cells were treated with siomycin A at the indicated doses for 48 hours. The cells were maintained in drug-free culture media for 15 days, and the cell colonies were stained with violet crystal. The cells were cleaved by 10% SDS, and cell viability was tested b7y spectrometer at the OD570 wavelength (5 repeats/sample). (b) CNE2TR cells were seeded in 24-well platea and treated with siomycin A at the doses shown for 24 hours. The cells were stained by Annex V/PI, and apoptotic cells were detected by flow cytometry.
