# Supplementary figures and images for: The FOXM1–ABCC5 axis contributes to paclitaxel resistance in nasopharyngeal carcinoma cells
Source: Cell Death Dis. 2017 Mar 9;8(3):e2659–. doi: 10.1038/cddis.2017.53 (PMC5386553; doi:10.1038/cddis.2017.53)

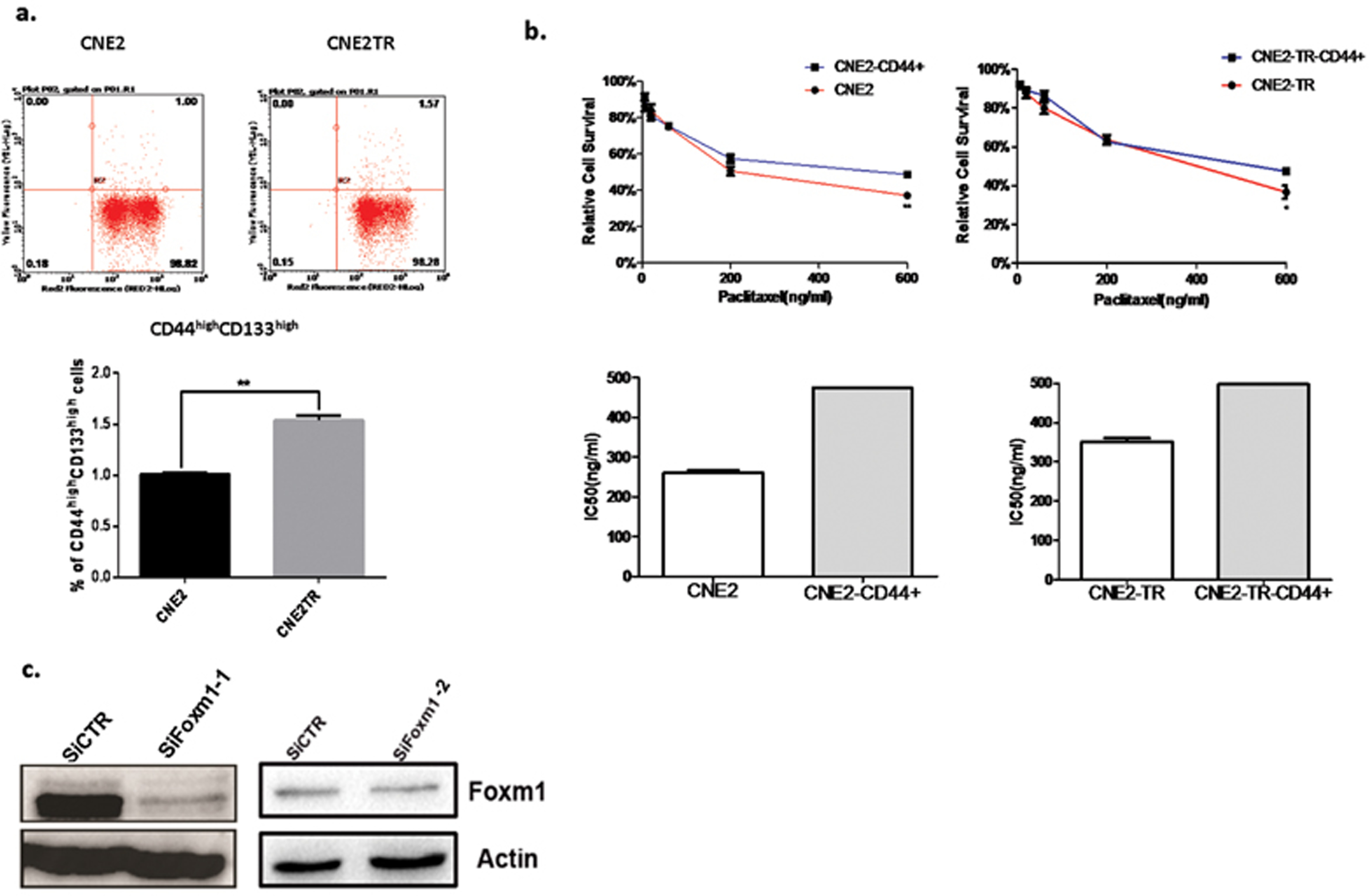

Supplement: Supplementary Figure S1 [file cddis201753x2.tif]

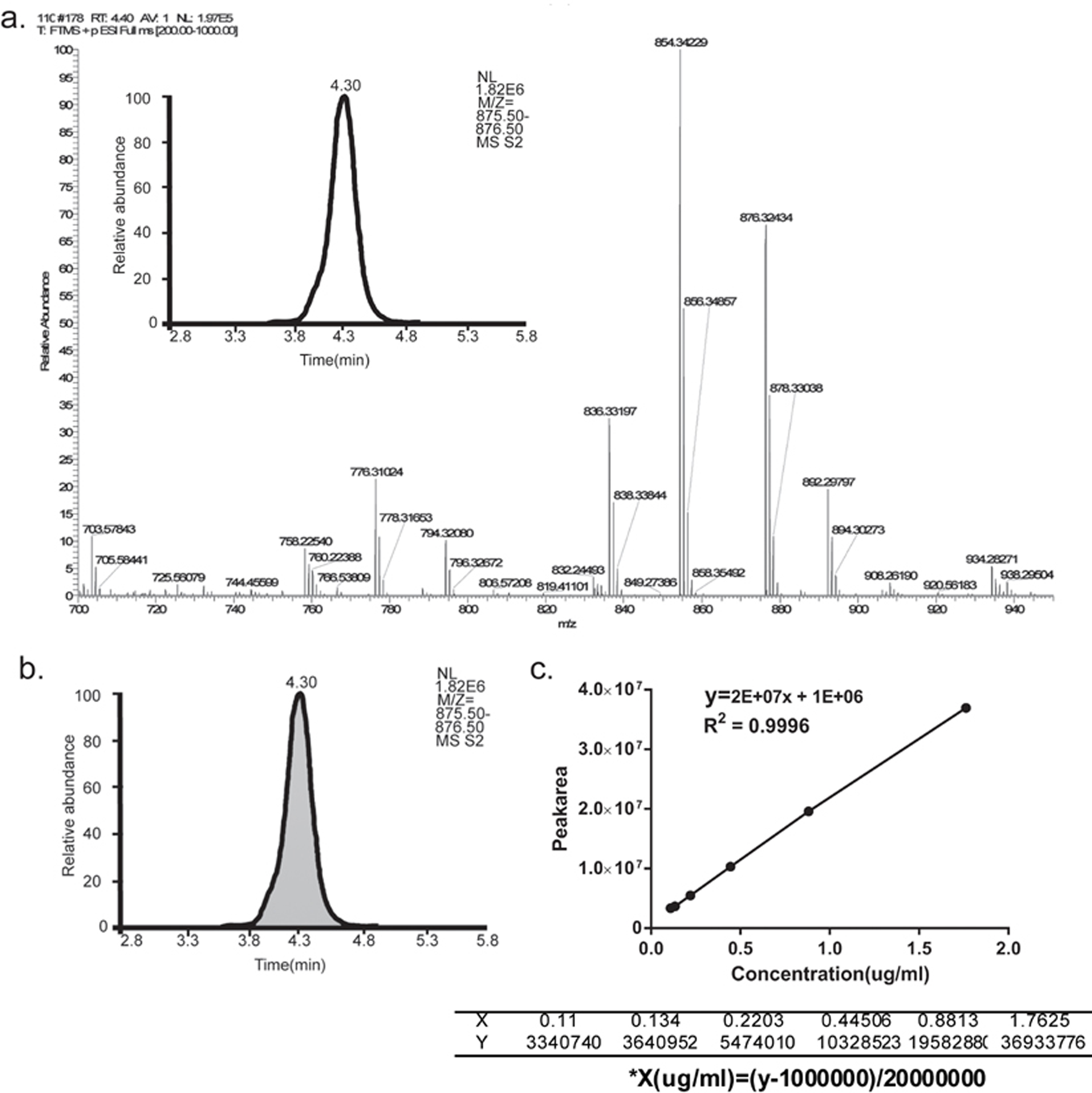

Supplement: Supplementary Figure S2 [file cddis201753x3.tif]

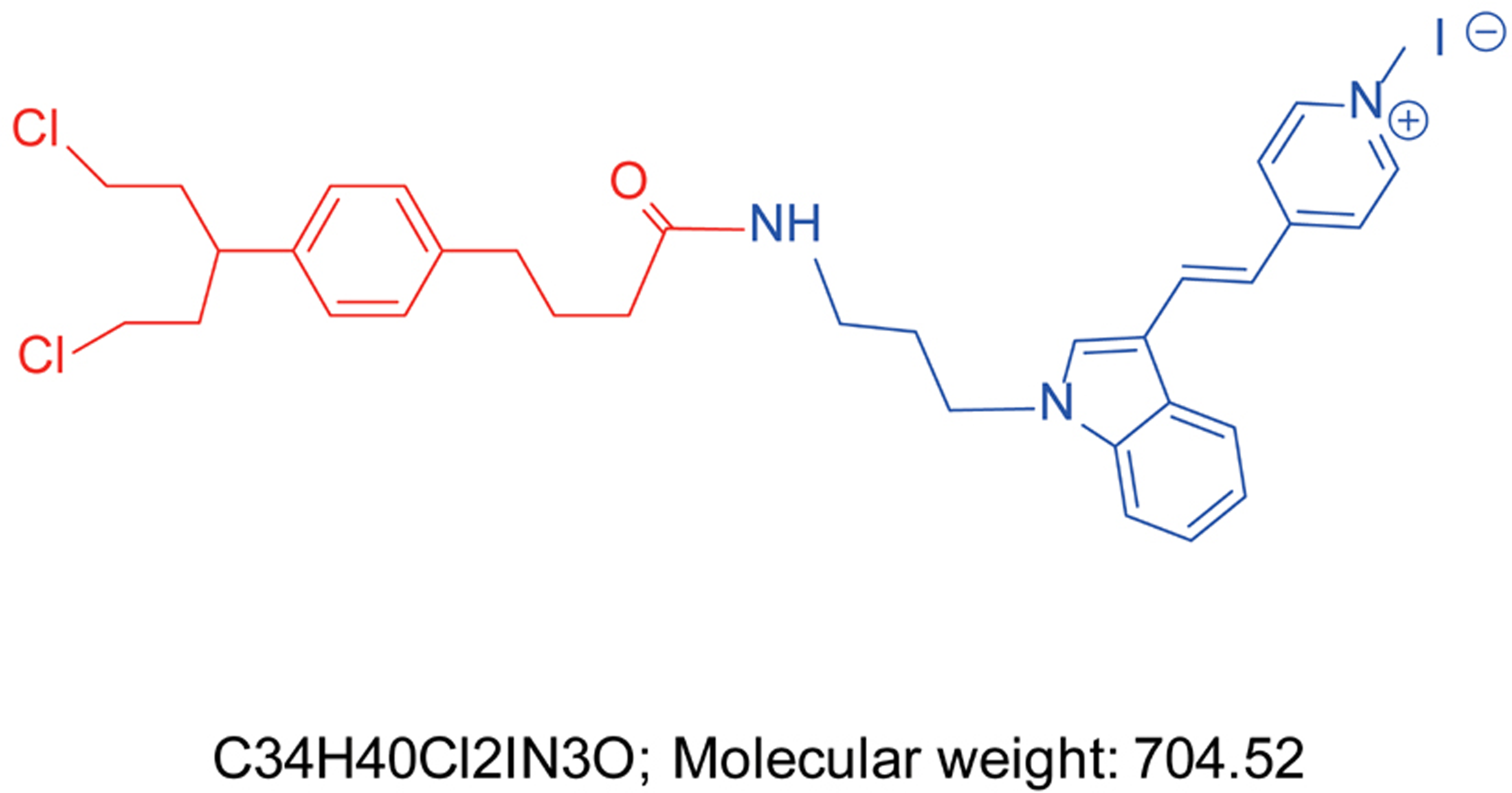

Supplement: Supplementary Figure S3 [file cddis201753x4.tif]

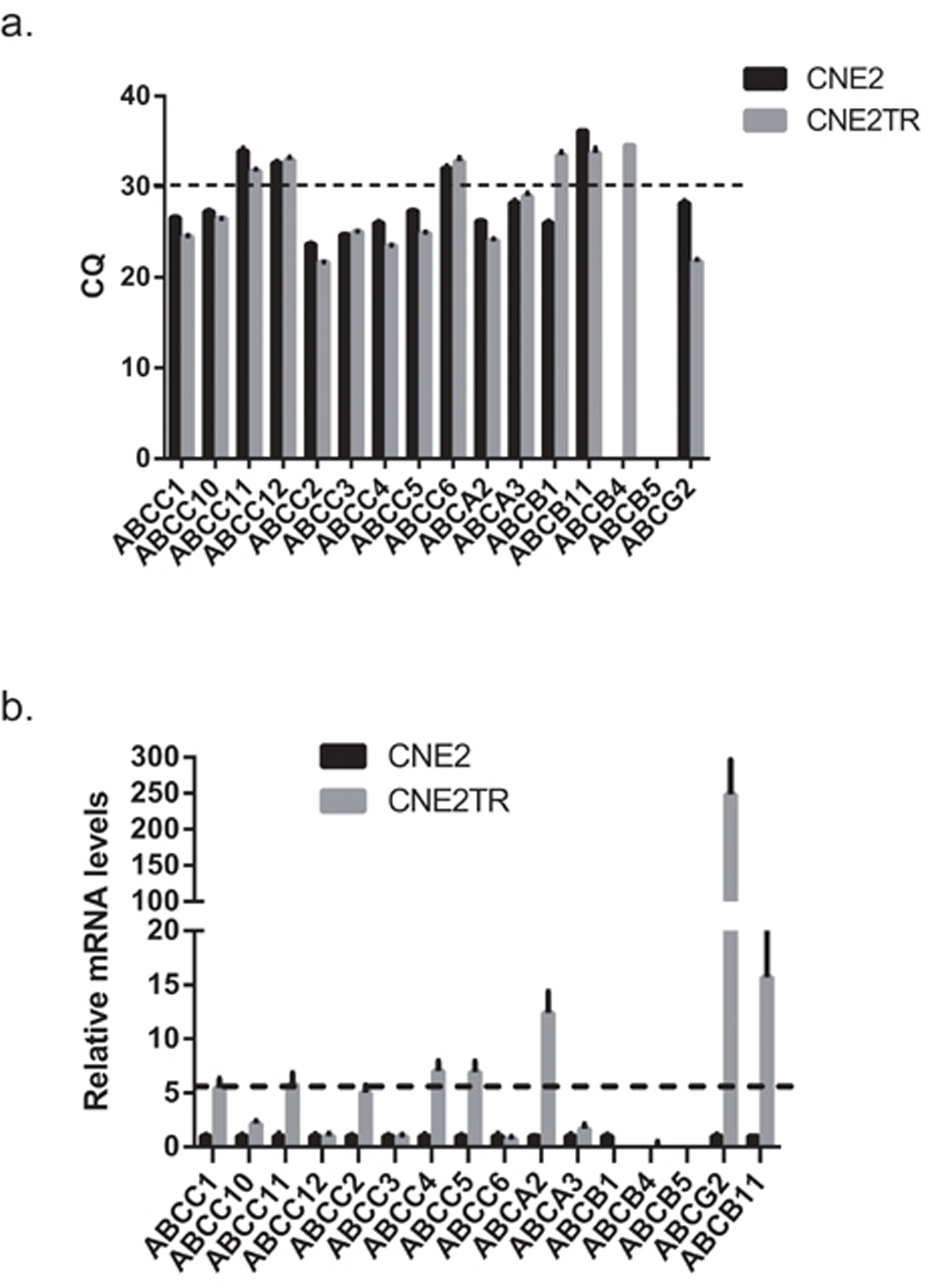

Supplement: Supplementary Figure S4 [file cddis201753x5.tif]

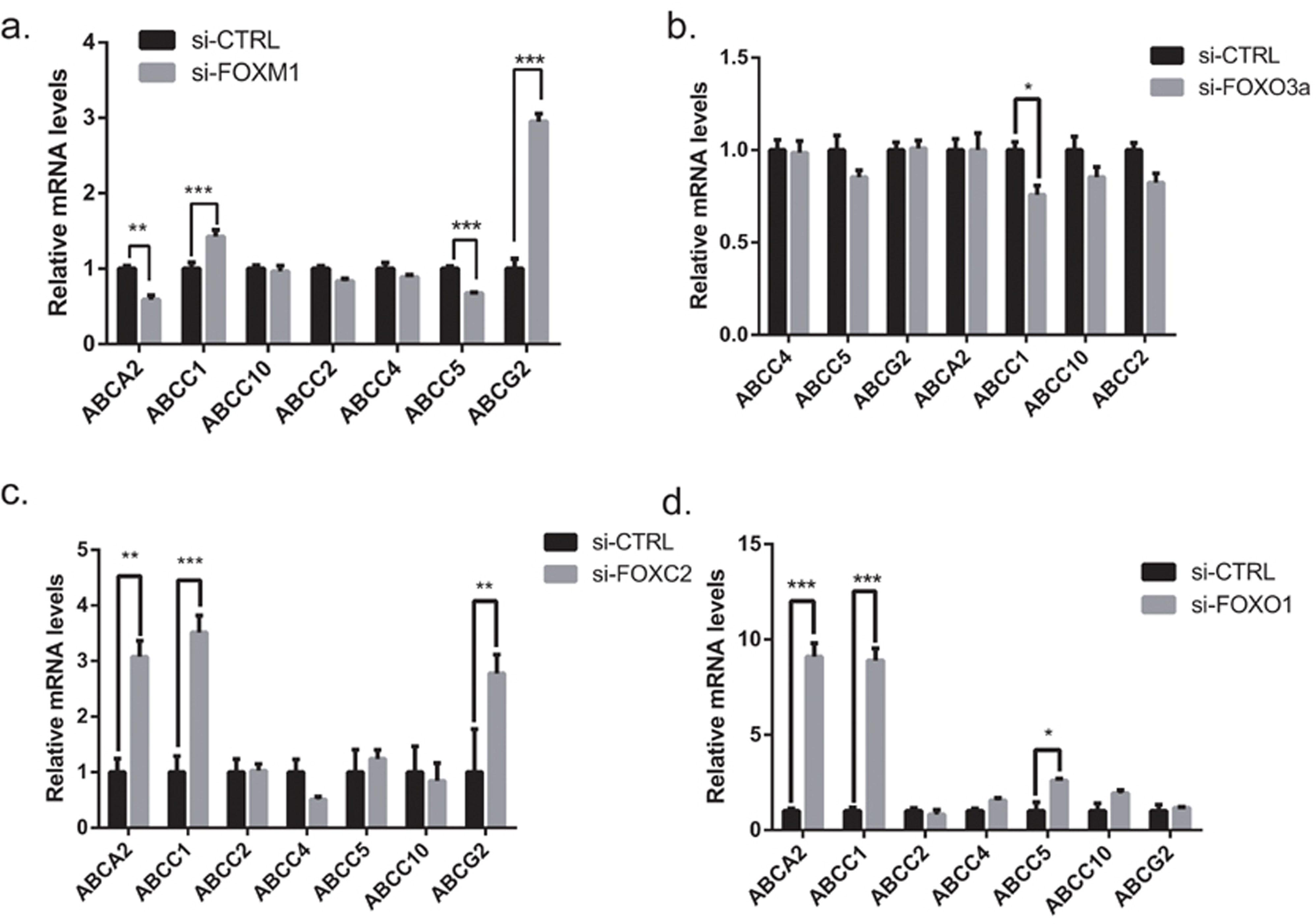

Supplement: Supplementary Figure S5 [file cddis201753x6.tif]

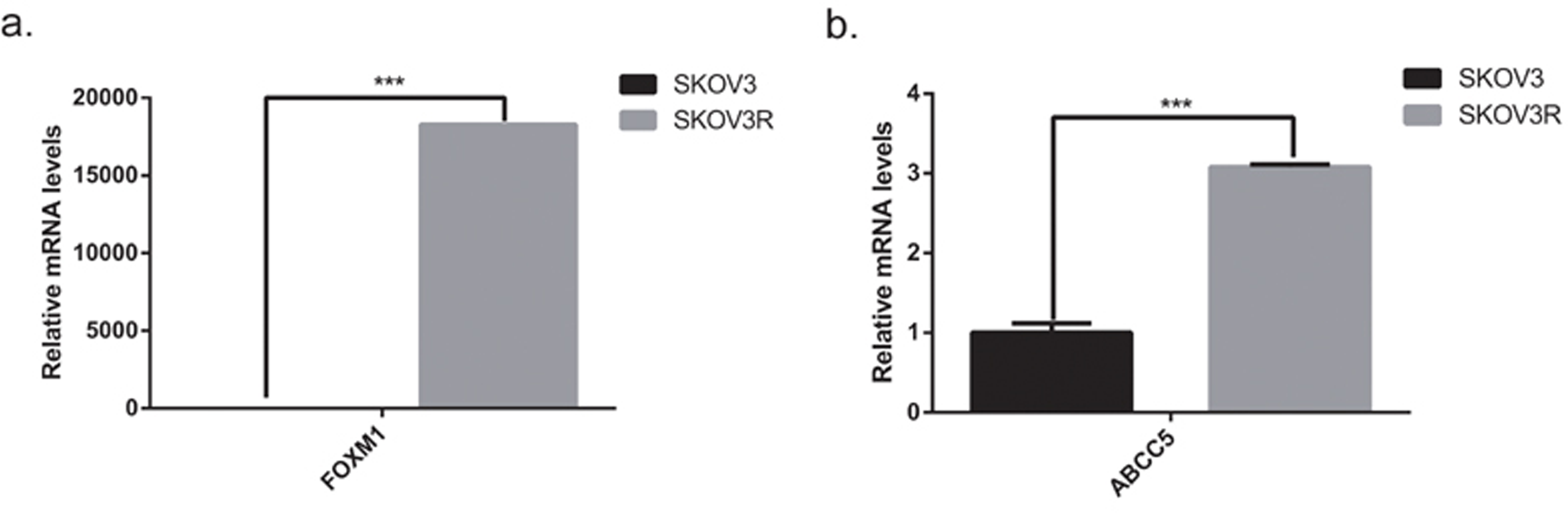

Supplement: Supplementary Figure S6 [file cddis201753x7.tif]

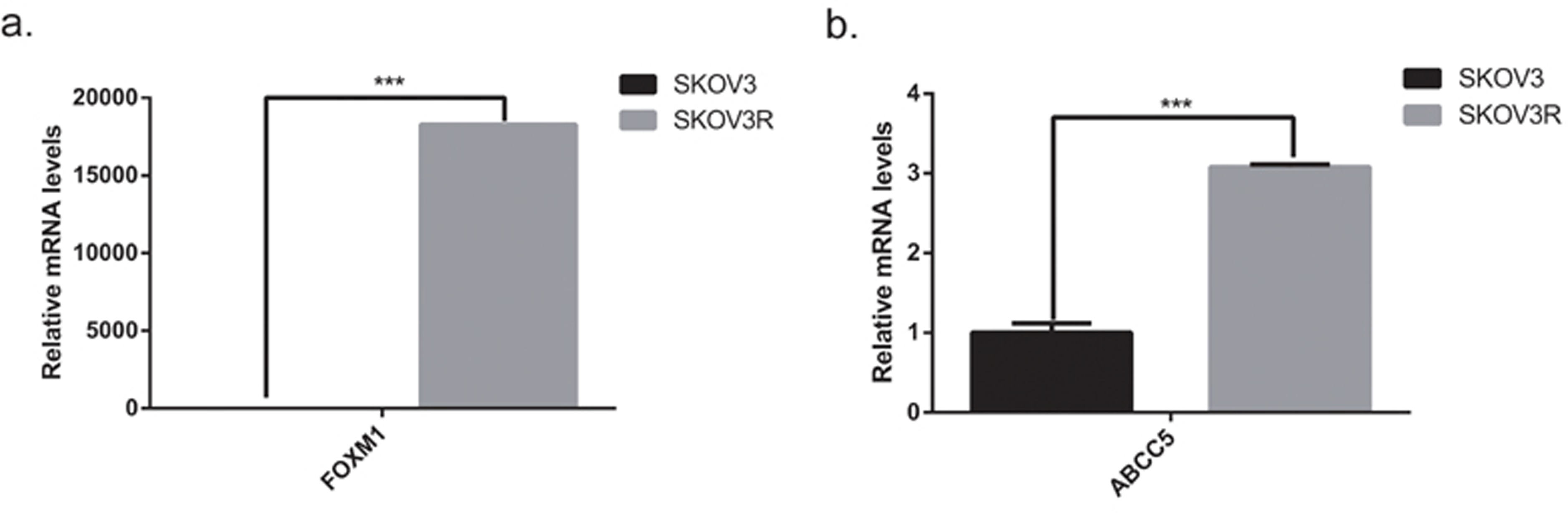

Supplement: Supplementary Figure S7 [file cddis201753x8.tif]
